# Tuning 4f‐Center Electron Structure by Schottky Defects for Catalyzing Li Diffusion to Achieve Long‐Term Dendrite‐Free Lithium Metal Battery

**DOI:** 10.1002/advs.202202244

**Published:** 2022-06-08

**Authors:** Jing Zhang, Rong He, Quan Zhuang, Xinjun Ma, Caiyin You, Qianqian Hao, Linge Li, Shuang Cheng, Li Lei, Bo Deng, Xifei Li, Hongzhen Lin, Jian Wang

**Affiliations:** ^1^ School of Materials Science and Engineering Xi'an University of Technology Xi'an 710048 China; ^2^ i‐Lab and CAS Key Laboratory of Nanophotonic Materials and Devices Suzhou Institute of Nano‐tech and Nano‐bionics Chinese Academy of Sciences Suzhou 215123 China; ^3^ Helmholtz Institute Ulm (HIU) Ulm D89081 Germany; ^4^ Inner Mongolia Key Laboratory of Carbon Nanomaterials Nano Innovation Institute (NII) College of Chemistry and Materials Science College of Mathematics and Physics Inner Mongolia Minzu University Tongliao 028000 China

**Keywords:** catalytic effect, dendrite‐free lithium, electronic redistribution, lithium metal batteries, Schottky defect modulation, sulfur cathodes

## Abstract

Lithium metal is considered as the most prospective electrode for next‐generation energy storage systems due to high capacity and the lowest potential. However, uncontrollable spatial growth of lithium dendrites and the crack of solid electrolyte interphase still hinder its application. Herein, Schottky defects are motivated to tune the 4f‐center electronic structures of catalysts to provide active sites to accelerate Li transport kinetics. As experimentally and theoretically confirmed, the electronic density is redistributed and affected by the Schottky defects, offering numerous active catalytic centers with stronger ion diffusion capability to guide the horizontal lithium deposition against dendrite growth. Consequently, the Li electrode with artificial electronic‐modulation layer remarkably decreases the barriers of desolvation, nucleation, and diffusion, extends the dendrite‐free plating lifespan up to 1200 h, and improves reversible Coulombic efficiency. With a simultaneous catalytic effect on the conversions of sulfur species at the cathodic side, the integrated Li–S full battery exhibits superior rate performance of 653 mA h g^−1^ at 5 C, high long‐life capacity retention of 81.4% at 3 C, and a high energy density of 2264 W h kg^−1^ based on sulfur in a pouch cell, showing the promising potential toward high‐safety and long‐cycling lithium metal batteries.

## Graphical abstract

The electronic structure of CeO_2_ is initially modulated by introducing Schottky defects, providing more active sites for interacting with lithium ion/atom. Higher Schottky defect concentration is more beneficial to motivate charge transfer effect on manipulating horizontal deposition, achieving uniform and smooth plating Li surface with long‐term cycling of 1200h and ultra‐low overpotentials.

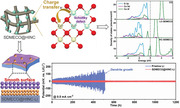

## 1 Introduction

Energy storage technologies are capable of greatly revolutionizing people's life to enjoy more convenient styles from portable smart electronics to emerging electrical vehicles.^[^ ^1^ ^]^ In comparison to commercial lithium ion battery based on graphite anode, rechargeable lithium metal anodes have attracted wide attentions in virtue of their high theoretical energy density (3860 mA h g^−1^) and the lowest electrochemical potential (−3.04 V vs SHE).^[^ ^2^ ^]^ However, the practical application of metallic lithium anode still faces rigorous challenges such as uncontrollable dendrites growth due to random dispersions of lithium ion/atom flux within the solvation surroundings, and cracking of the fragile solid electrolyte interphase (SEI) as a result of large volumetric changes.^[^ ^3^ ^]^ More severely, the repeated formation of fresh SEI continuously consumes and eventually exhausts the little finite electrolyte,^[^ ^4^ ^]^ resulting in low Coulombic efficiency (CE), limited lifespan, and even safety hazards by short circuiting.^[^ ^5^ ^]^

Up to date, diverse attempts have been taken to handle the aforementioned issues.^[^ ^6^ ^]^ Constructing porous conductive hosts are popular strategies used for accommodating lithium with greatly reduced deposition current density for smooth plating.^[^ ^7^ ^]^ However, the relatively high weight hosting matrix inevitably sacrifices the entire energy density of the electrodes. An alternative choice is covering the metallic lithium surface with light‐weight artificial conductive layers such as nanocarbon, alloy, and MXene to improve the wettability/affinity to lithium atoms/ions.^[^ ^1^, ^7^, ^8^ ^]^ Thermodynamically, the freshly reduced Li atoms tend to migrate through the artificial conductive layer and deposit on the beneath metallic lithium rather than being directly localized on the outer surface.^[^ ^9^ ^]^ Different from popular architecture designs, little attention has been paid to manipulate the intrinsic nucleation and diffusion kinetics of the atomic lithium inflow on the Li interface.^[^ ^2^, ^3^, ^5^, ^7^, ^10^ ^]^ As stated above, Li striping/plating behaviors depend greatly on the interfacial physicochemical properties. From a fundamental aspect, the lithium process contains multi‐steps including the dissociation of solvated Li ions to bare ones, lithium atom formation after coupling with electrons, and the diffusion of the formed lithium atoms.^[^ ^11^ ^]^ These steps suffer from huge barriers such as desolvation barrier, nucleation barrier, and ionic/atomic diffusion barriers.^[^ ^12^ ^]^ Based on the classical Arrhenius formula $k=Ae^{-(E_{a}/\mathrm{RT})}$, decreasing these barriers can help improve the lithium plating kinetics toward dendrite‐free morphologies.^[^ ^4^, ^7^, ^9^ ^]^ Therefore, the core challenge is concentrated on looking for proper catalysts/activators to speed up the desired lithium kinetics.

As well known, the electronic structures and chemical surroundings of the catalysts have a close connection with the catalytic capability.^[^ ^13^ ^]^ Our previous studies had disclosed 3d‐center electronic structures play an essential role in catalyzing the polysulfide and lithium sulfide conversion reactions.^[^ ^14^ ^]^ These findings inspire us to modulate the electronic density distribution in catalysts to increase their activities. Defect engineering is a feasible way to redistribute the internal electrons and bring about intrinsically active sites or synergistic locates.^[^ ^15^ ^]^ Schottky defects may be useful to regulate the electronic density in transition metal oxides to render more active sites to decrease the nucleation and diffusion barriers of lithium ion/atom.^[^ ^16^ ^]^ Compared with engineering of 3d‐center electrons, tuning the 4f‐center electrons is more facile and may potentially render higher activity to the catalyst due to the unsaturated 4f orbits.^[^ ^17^ ^]^ As far as we know, tuning the 4f‐electrons of metal compound catalysts to modulate lithium plating kinetics has never been reported. The defect‐induced electronic structure reconstruction is also deemed to promote the overall plating electrochemistry through affecting the active nucleation sites.

**Figure 1:**
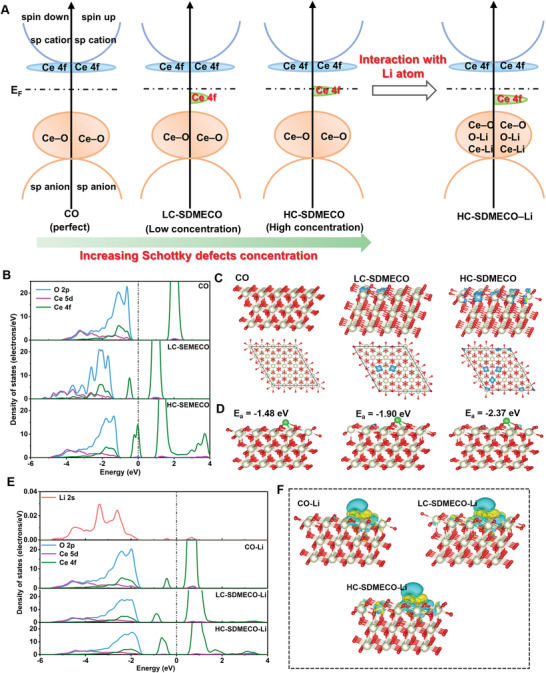
Simulation of the Schottky defects in modulating electronic structure with/without Li atom. A) Schematic explanation of the 4f electron modulation strategy by increasing the Schottky defects concentration, and the subsequently electron transfer interaction of the modulated structure with Li atom; B) the projected density of states (PDOS) simulated from the surface atoms in the unabsorbed configurations of CO, LC‐SDMECO, and HC‐SDMECO; C) the front and top views of spin density plots for the three unabsorbed configurations (the white and red balls represent the Ce and O atoms, respectively; and the light bule isosurfaces represent the spin‐up for Ce 4f electrons); D) adsorption configurations of Li on CO, LC‐SDMECO, and HC‐SDMECO (the green ball represent the Li atom and the deleted oxygen atoms for constructing the Schottky defects are depicted as cyan balls); E) the PDOS of Li 2s, O 2p, Ce 5d, and Ce 4f from the surface atoms in CO‐Li, LC‐SDMECO‐Li, and HC‐SDMECO‐Li and F) the corresponding charge density difference of the three models (cyan and yellow isosurfaces represent losing and gaining electrons, respectively.)

Herein, the 4f‐center electron state structures are regulated to dramatically increase catalytic activity through Schottky defects, realizing dendrite‐free and long lifespan Li metal battery. Briefly, as simulated by density functional theory in **Figure** **1**A, the electronic densities modulated by Schottky defects are redistributed in CeO_2_ (CO) within highly‐conductive and interconnected N‐doped carbon nanotubes networks (SDMECO@HINC), generating large number of active sites. Consequently, the SDMECO@HINC exhibits superior electrocatalytic activity in propelling the desolvation and Li diffusion kinetics, achieving smooth dendrite‐free lithium plating. Serving as modulators on lithium electrodes (SDMECO@HINC‐Li), a long lifespan (1200 h at 0.5 mA cm^−2^), and high Coulombic efficiency (98% after 100 cycles) are achieved without dendrite formation. Also, the SDMECO@HINC hybrid effectively catalyzes the conversion reactions of sulfur species. The so‐fabricated Li–S full cells based on SDMECO@HINC deliver superior rate performance of 653 mA h g^−1^ at 5 C and high capacity retention of 81.4% at 3 C after 500 cycles, signifying great promise of SDMECO@HINC for practical application.

## 2 Results and Discussion

### 2.1 Theoretical Investigation of Schottky Defects Motivated 4f Electronic Structure with Li Atoms

Access to the desired electrocatalytic properties in propelling the Li deposition kinetic behaviors require an in‐depth understanding of electronic structure evolutions before and after interacting with Li atoms. As inspired by the intrinsic electron adjustability, the electronic band structure of the CeO_2_ is expected to highly dependent on the introduction of Schottky defects due to the 4f‐band state shift with the increase of Schottky defects concentration (Figure 1A). The 4f‐band state presents a remarkable recover through charge transfer by interacting with Li atom, which plays a key role in the uniform capture of Li atom in the initial nucleation process. In detail, to verify the feasibility of Schottky defects in modulating electronic densities to uniformize the Li nucleation, projected density of states (PDOS), charge density difference (CDD), spin density, and energy evaluation were performed. The low and high Schottky defects concentration in CeO_2_ are simulated, named as LC‐SDMECO and HC‐SDMECO, respectively. As displayed in Figure 1B, the PDOS states moving toward the Fermi level are exhibited in LC‐SDMECO and HC‐SDMECO, evidencing the adjustability and orbital hybridization of 4f electron structure through introduction of Schottky defects. Meanwhile, the Fermi level state is strengthened in HC‐SDMECO and the corresponding spin polarity originating from Ce‐4f electrons is significantly enhanced (Figure 1B,C).

As expected in Figure 1D and Figure S1, Supporting Information, the adsorption energies increase along with the strength of electronic modulation, suggesting the higher Schottky defect concentration is more favorable for Li capture and uniform Li nucleation. The PDOS show the apparent hybridizations between Li 2s and O 2p, Ce 5d, and Ce 4f orbitals (Figure 1E), forming the Ce—Li and O—Li bonds. The Ce—Li bond for the LC‐SDMECO and HC‐SDMECO might be weakened due to the obviously reduction of Ce‐4f electrons that participate in bonding, indicating core roles of Ce‐4f electrons in modulating Li atom. Owing to the debilitating hybridization between the electronic orbitals of Ce and O atoms, the intensity of O—Li bond is enhanced indirectly by oxygen vacancies. On the other hand, the oxygen defects would enhance the adsorption ability toward Li through destroying the charge balance of perfect CeO_2_ (111) surface for forming an electric field. Such electronic transfers between the SDMECO and Li atom can also be reflected by the CDD in Figure 1F.

### 2.2 Synthesis and Morphology Characterizations of Schottky Defects in SDMECO@HINC

**Figure 2:**
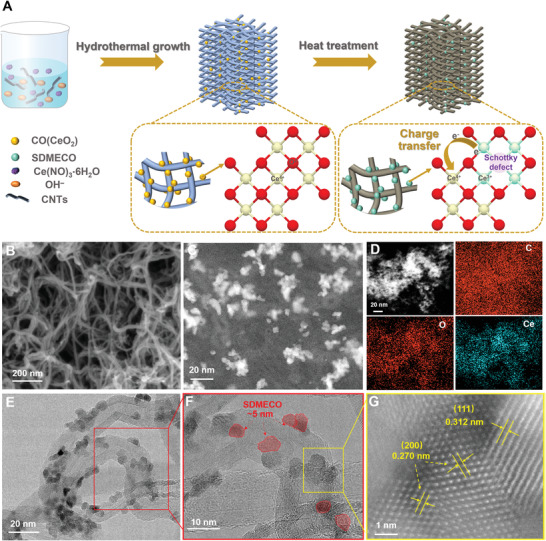
Schematic synthesis illustration and morphology of the SDMECO@HINC nanocomposite. A) Schematic illustration of the synthesis procedure of the SDMECO@HINC; B) SEM image; C) STEM image; D) EDX elemental maps; E,F) TEM images; G) HRTEM image of SDMECO@HINC.

Encouraged by these simulation results, the SDMECO@HINC nanocatalysts was synthesized by combining the hydrothermal reaction with hydrogen treatment, as depicted in **Figure** **2**A. Briefly, the fabricated CeO_2_ anchored highly‐conductive and interconnected carbon nanotube networks (CO@HINC) was initially synthesized from the solution precursor by hydrothermal reaction, which was further annealed under reducing atmosphere (Ar/H_2_ in volume ratio of 5%:95%) for removing a certain amount of oxygen atoms to form Schottky defects to modulate electron structure. The structure and morphology characteristics of the as‐synthesized SDMECO@HINC were then carried out as detailed verification. The scanning electron microscopy (SEM) and transmission electron microscopy (TEM) images of the as‐prepared SDMECO@HINC are shown in Figure 2B–G. The SEM images in Figure 2B and Figure S2, Supporting Information show the nanoscale porous interconnected morphologies of SDMECO@HINC, CO@HINC, and HINC, respectively. Such conductive network can beneficially facilitate the fast electron/ion transport and guarantee sufficient electrolyte infiltration into the electrode during the stripping/plating electrochemistry. Even treated under an Ar/H_2_ atmosphere, the nanostructured morphology of nanoparticles is still reserved in contrast to CO@HINC (Figure S1B, Supporting Information). Further recorded by the scanning TEM (STEM) image in Figure 2C, many bright species evenly blooming on the HINC surface are discerned as SDMECO (the higher‐density or heavier elements of Ce generate brighter contrast due to the mass thickness contrast differences), indicating the well‐grown and uniform distributed SDMECO nanoparticles on the HINC without any large aggregations. Energy dispersive X‐ray (EDX) mapping images also confirm that the elemental Ce, O, and C are evenly scattered within the nanocomposite (Figure 2D). The TEM images (Figure 2E,F) present that a clearer size of the SDMECO nanoparticles closely attached on the conductive carbon is as smaller as about 5 nm in diameter, which are highlighted by the red shades. At the same time, the high‐resolution STEM image in Figure 2G reveals the typical atomic structure and the interplanar spacings of lattice fingerprint of the nanoparticle are estimated to be 0.270 and 0.312 nm, similar to the (200) and (111) spacing of cerium oxide, respectively.

**Figure 3:**
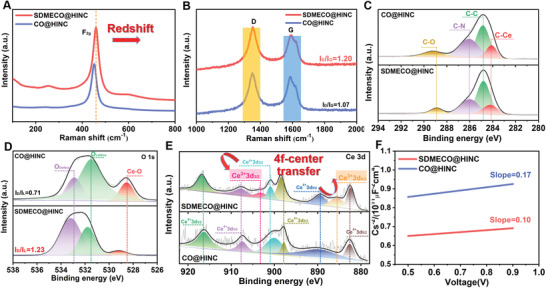
Characterizations of electronic reconstruction in the SDMECO@HINC. Raman Comparisons of A) F_2g_ characteristic peak and B) D and G peaks in the SDMECO@HINC and CO@HINC nanocomposites, respectively; the high‐resolution XPS spectra comparison of C) C 1s; D) O 1s and E) Ce 3d between the SDMECO@HINC and CO@HINC; F) Mott−Schottky plots obtained on defective SDMECO@HINC and CO@HINC symmetric cells with 1 mol L^−1^ Na_2_SO_4_ electrolyte at 1 kHz frequency.

In the consecutive structural characterization steps, the resultant characteristic diffraction peaks located at 28.6°, 47.5°, and 56.3° in the X‐ray diffraction (XRD) patterns (Figure S3, Supporting Information) could be perfectly assigned to the fluorite cubic cerium oxide (JCPSD No. 34‐0394). The electronic structure variation in the as‐synthesized SDMECO@HINC nanocomposite is confirmed by Raman spectra, X‐ray photoelectronic spectroscopy (XPS) and electrochemical impedance spectroscopy (EIS), as presented in **Figure** **3**. As displayed in Figure 3A, the peak around 464 cm^−1^ assigned to feature F_2g_ is ascribed to fluorite lattice structure of the symmetric stretch mode of Ce‐O_8_ crystal unit.^[^ ^18^ ^]^ After introducing Schottky defects in the CeO_2_, an observed redshift of the F_2g_ peak is created, implying the partial oxygen atoms in the crystal are removed and the electronic density distribution is modulated. Also, under the reduction atmosphere, more defects are formed in the nanocarbon with catalytic activity as suggested by the fact that the *I*_D_/*I*_G_ increases from 1.07 for CO@HINC to 1.20 for SDMECO@HINC (Figure 3B), indicating the electronic density variation in the carbon atom surrounding.^[^ ^13^, ^19^ ^]^

To further clearly read the Schottky defects and electronic interaction, XPS is employed to inspect the electronic changes of the metallic center and the valence states (Figure 3C–E; Figure S4, Supporting Information). The feature peaks of Ce 3d, C 1s, N 1s, and O 1s are clearly observed in the two samples, as presented in Figure S4, Supporting Information. The peaks in the high‐resolution C 1s spectrum located at 284.7, 286.0, and 288.9 eV are assigned to C—C, C—N, and C—O species, respectively (Figure 3C).^[^ ^20^ ^]^ The existence of C—Ce (284.1 eV) bond is supposed to the formation of heterojunction interfaces between the HINC and SDMECO, which significantly enhances the physical electronic contact and promotes catalytic activity through speeding up the charge transfer for fast lithium plating. At the same time, in the high‐resolution O 1s spectra in Figure 3D, two peaks at 531.5 (*I*_L_) and 533.2 eV (*I*_D_) are attributed to the contributions from lattice oxygen and defective oxygen in the metal oxide,^[^ ^13a^ ^]^ respectively. More oxygen defects are liberated in SDMECO@HINC due to the increased peak intensity ratio of *I*_D_/*I*_L_ than the intact CO@HINC (1.23 vs 0.71). The valence state of oxygen strongly influences the defect caused electronic redistribution. Along with the typifying O 1s, the variational valence state of Ce is also detected after erasure of partial oxygen atoms. Compared with perfect CO@HINC, the defect‐rich structure shows obviously positive shift of both the Ce^4+^ 3d_3/2_ and Ce^4+^ 3d_5/2_ peaks around 900.2 and 897.6 eV (Figure 3E), respectively, indicating the variation of coordination surroundings with the change of electron density. Meanwhile, the new embossments of the Ce^3+^ 3d_3/2_ and Ce^3+^ 3d_5/2_ peaks at 885.5 and 903.3 eV in the high‐resolution Ce 3d spectrum are ascribed to the reduction from Ce^4+^ to Ce^3+^ with the aim of maintaining the electric neutrality.^[^ ^17^ ^]^ Overall, the co‐occurred changes of these typical peaks are direct proofs to the 4f‐center electron density transfer inducing valence band restructuring upon the inception of the Schottky defects in the so‐synthesized SDMECO@HINC.^[^ ^13^, ^21^ ^]^ Then, Mott−Schottky plots are depicted through EIS measurements to reveal the presence and roles of Schottky defects on the intrinsic properties of the nanocomposites. It is found that both the SDMECO@HINC and CO@HINC samples display n‐typed semiconductor nature (Figure 3F). According to Mott−Schottky formula ($N_{d}=\frac{2}{e\varepsilon \varepsilon_{0}}\cdot \frac{dV}{d(1/C^{2})}$), the smaller slope of SDMECO@HINC implies higher donor density (*N*_d_) than that of CO@HINC, which discloses the larger amount of Schottky defects in SDMECO.^[^ ^14a^ ^]^

### 2.3 Dendrite‐Free Li Plating/Stripping Performances on 4f Electron Layer Regulated Li Anodes

**Figure 4:**
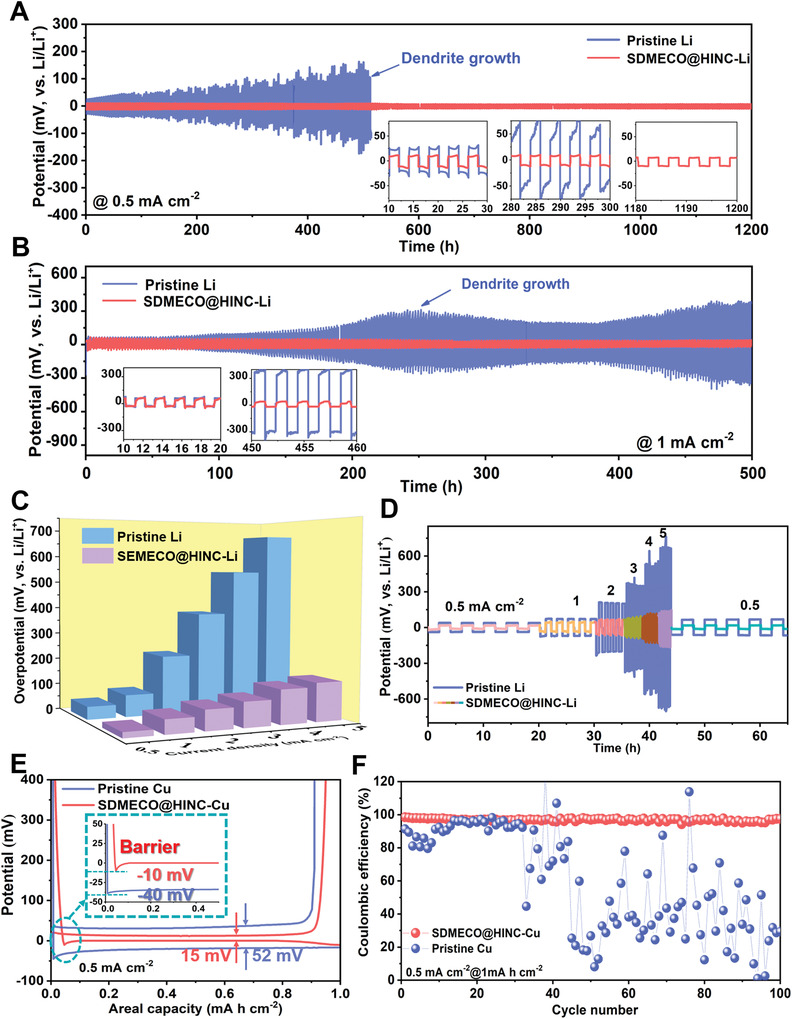
Li plating/stripping behaviors affected by 4f electron‐regulated activator on Li anodes. Galvanostatic cycling of symmetric cells depending on the defect induced SDMECO@HINC‐Li and pristine Li electrodes under stripping/plating capacity of 1 mA h cm^−2^ A) at current rate of 0.5 mA cm^−2^ and B) at current rate of 1 mA cm^−2^; C) overpotentials and D) the galvanostatic stripping/plating profiles comparison between the two symmetric cells cycled under shifted current densities with the capacity of 1 mA h cm^−2^; E) voltage profiles comparison of pristine Cu and SDMECO @HINC‐Cu electrodes in first cycle planting 1 mA h cm^−2^ capacity of Li at 1 mA cm^−2^ (the inset comparison curve of the Li nucleation barriers on different electrodes in asymmetric cell) and F) the corresponding coulombic efficiencies of Cu–Li cells based on the pristine Cu and SDMECO@HINC‐Cu electrodes within 100 cycles.

In order to fulfill the target of long‐life for large‐scale storage applications, it is of remarkable significance for rechargeable lithium metal batteries to maintain reversible performances. The lifespan of the batteries is highly associated with the anodic Li dendrite formation during electrodeposition. To manifest the electronic effects of Schottky defected SDMECO@HINC on modulating the plating/stripping lithium behaviors, the symmetric/asymmetric cells based on the lithium electrodes with/without SDMECO@HINC modulation (denoted as SDMECO@HINC‐Li and pristine Li, respectively) were assembled and evaluated at designed current densities under 1 mA h cm^−2^. As displayed in the EIS of Figure S5A, Supporting Information, the modified SDMECO@HINC‐Li electrode shows a decreased charge transfer resistance than the controlled one (9 vs 26 Ω), implying faster charge exchange across the interface after 4f‐electron modulation. Plating at 0.5 mA cm^−2^, the initial Li atom nucleation barriers on the pristine Li is about 19 mV and decreased to 11 mV when reaching the catalytic active surface of SDMECO@HINC (Figure S5B, Supporting Information), indicating the feasibility of fast desolvation and nucleation in Figures S6,S7, Supporting Information. As indicated in Figure S7A,B, Supporting Information, the initial lithium is vertically plated as small needles on pristine Li surface and tend to continuously grow up. In a stark contrast, with the aid of the upper catalytic modulation layer, the Li atom plating is manipulated by the Schottky defect‐rich SDMECO@HINC layer to start a planar deposition and a continuous layer is formed with the increase of plating amount of Li (Figure S7C,D, Supporting Information). In further cycling, the potentials of pristine Li show a continuously increasing tendency and finally become short circuit after almost 500 h. In sharp contrast, with the catalytic modulation of SDMECO@HINC on the metallic lithium surface, the diffusion and plating behaviors of lithium atoms seem much easier and the overpotentials stabilized for ≈13 mV (**Figure** **4**A). Meanwhile, as highlighted in the magnitude insert figures, the SDMECO@HINC‐Li electrode is capable of lasting for 1200 h with the steady overpotentials without dendrite formation. Operating the current density up to 1 mA cm^−2^, the SDMECO@HINC‐Li electrode still stabilizes ultralow overpotentials around 50 mV and survives for 500 h (Figure 4B), significantly better than that of pristine one. In comparison, Figure S8, Supporting Information displays that the HINC‐Li||HINC‐Li cell exhibited an inferior performance in lithium plating/stripping behaviors to that with SDMECO catalyst, strongly suggesting that the stable and high performance of the metallic Li should be attributed to the modulation effect of SDMECO. Even with plating capacity of 2 mA h cm^−2^, the SDMECO@HINC‐Li electrode retains a low overpotentials no more than 100 mV within 700 h at 2 mA cm^−2^ (Figure S9, Supporting Information). These enhanced cycling plating/stripping behaviors directly elucidate the superb capability of the catalytic SDMECO@HINC on modulating lithium ion/atom diffusion, realizing the suppression of lithium dendrite growth. With progressively increasing current density from 0.5 to 5 mA cm^−2^, the overpotentials of the SDMECO@HINC‐Li electrode and pristine Li electrode are summarized and displayed in Figure 4C,D. Enhancing rate current density from 3 to 5 mA cm^−2^, the overpotential of the SDMECO@HINC‐Li electrode only increases by about 40 mV. Even at 5 mA cm^−2^, the SDMECO@HINC‐Li electrode still outputs superior low overpotential merely around 145 mV and excellent stability, which indicates the substantially improved lithium kinetics and reversibility in comparison to the control one up to 700 mV (Figure 4C,D). The above results have clearly revealed that the higher overpotentials are more liable to trigger the Li dendrite formation during Li electrodeposition with locally high current densities. In the consecutive steps, solvated Li species are hard to form bare Li ions in time and failed to diffuse timely, which results in the uneven surface and finally the formation of Li dendrites.

On the other hand, Coulombic efficiency (CE) is known as another evaluation criterion to inspect the reversibility of the Li plating behaviors, as illustrated in Figure 4E,F and Figure S10, Supporting Information. The CE is defined as the stripping capacity divided by plating capacity. Obviously, the initial Li atom nucleation barrier is decreased from 40 mV for pristine Cu to 10 mV for the SDMECO@HINC‐Cu electrode (inset of Figure 4E), indicating the lithiophilic affinity ability toward the lithium nucleation. And the initial voltage curve of SDMECO@HINC‐Cu electrode in Figure 4E further displays a smooth voltage dip with an ultralow overpotential gap of only 15 mV at 0.5 mA cm^−2^, nearly four times lower than that of the bare Cu substrate (52 mV). This significantly decreased overpotential implies the much‐boosted affinity to lithium atoms in virtue of the 4f‐electronic modulation of SDMECO during the electrodeposition. In the successive cycling, the asymmetric cell based on pristine Cu undergoes a fluctuating CE tendency with a limited lifespan less than 40 cycles and then decayed to a failure state. However, the asymmetric cell based on SDMECO@HINC‐Cu stabilizes the CE over 98% even after operating for 100 cycles (Figure 4F; Figure S10, Supporting Information), much higher than that in the HINC, indicating a negligible contribution to the CE improvement of the HINC (Figure S11, Supporting Information). These results have comprehensively certified the aspiring manipulation of adjusting electronic state density to form more catalytic sites is suitable for fast electronic/ionic exchange with uniform Li plating/stripping behaviors to suppress the dendrite formation, achieving long‐life Li metal anode with robust current leap adaptability and high Coulombic efficiency.

### 2.4 Morphologies and Mechanism of SDMECO@HINC in Manipulating Lateral Lithium Plating Behaviors

**Figure 5:**
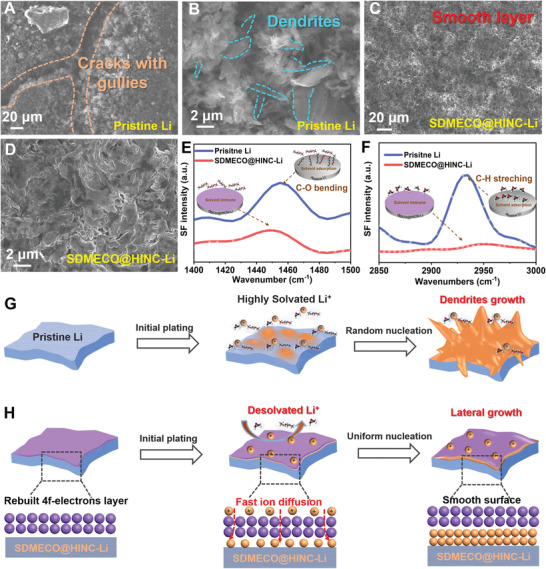
Schematic mechanism illustration of SDMECO@HINC activator in manipulating kinetics in lithium plating. A,B) The top view SEM images (taking samples from the symmetric cells at the end of the cycling); C,D) the top view SEM images of the exposed plated Li surface after uncovering the upper SDMECO@HINC layer; in situ SFG spectra comparison of the two lithium electrodes with E) DME/electrode interface and F) DOL/electrode interface, respectively; the schematic illustration of lithium plating behaviors on G) pristine Li and H) SDMECO@HINC‐Li from the initial plating to in the successive cycles.

The final cycled surface morphologies of the lithium foils with/without SDMECO@HINC modulation layer are also investigated by SEM, as shown in **Figure** **5**A–D and Figure S12, Supporting Information. Obvious cracks with gullies highlighted by the orange dot line are shown on the final cycled surface of the pristine Li surface (Figure 5A). In the high‐resolution SEM image, the needle‐like morphology on the upper surface of the electrode is captured as the generation of Li dendrites (Figure 5B). Such needle tips act as the charge aggregation centers in the subsequent reactions to trigger the cusp effect, which brings about the charge accumulation and accelerates Li deposition along the direction parallel to the needle, stimulating the further growth of Li dendrite. On the other hand, the brittle Li dendrites fracture from the root to form dead crystals contribute a lot to the electrode pulverization and volume expansion, which is responsible for the voltage degradation and even battery failure during cycling. According to the cross‐sectional SEM in Figure S12A, Supporting Information, the corresponding volume expansion ratio of the cycled pristine Li electrode including dead Li is roughly estimated as 31.5%. Eventually, a uniform and smooth distribution morphology on the metallic lithium surface without emergence of Li dendrites in the low‐ or high‐resolution SEM images is observed (Figure 5C,D). In the cross‐sectional SEM image, the active reacted lithium layer is only about 55 µm, showing the robust reversibility of the plated lithium atom (Figure S12B, Supporting Information). To the best of our knowledge, the morphology of the plating lithium atoms is also closely in connection with the generation of the bare Li ions from solvated surrounding on the interface. That is to say, the faster kinetics of the desolvation to form bare Li ions, the lower the barriers for the nucleation and plating will be. The corresponding interface information is further verified by means of the interface‐sensitive in situ sum frequency generation (SFG) spectroscopy. As shown in Figure 5E,F, the peak signals in the SFG spectroscopy around 1450 and 2930 cm^−1^ are assigned to C—O bending vibration signals of 1,2‐dimethoxyethane (DME) and C—H stretching of 1,3‐dioxolane (DOL), respectively. The higher and stronger SFG intensities of C—O and C—H on pristine Li surface than that on the SDMECO@HINC‐Li strongly demonstrate that the solvents of DME/DOL are high affinitive to pristine Li surface, prohibiting the smooth access of the subsequent Li^+^ ions and eventually resulting in non‐uniform nucleation (Figure S6A, Supporting Information). Thanks to the electronic interaction between the solvated Li^+^ and the defect‐based catalyst, the Li^+^‐solvent binding will be dramatically weakened, leading to easier desolvation. Thereby, the surrounding solvents are hardly adsorbed on the catalytic SDMECO@HINC layer, which effectively accelerates the desolvation process to generate free lithium ions from the solvated Li^+^ to realize uniform plating (Figure S6B, Supporting Information).

Combining the afore‐mentioned electrochemical analysis, the SEM morphologies, SFG with simulation results, the beneficial functions of 4f‐electronic structure by Schottky defects to provide more catalytic sites in SDMECO@HINC layer for guiding lithium kinetics for lithium deposition are also schematically depicted in Figure 5G,H. As exhibited in Figure 5G, the high affinity of the solvents around the Li ion would incur higher desolvation energy barrier, so that the solvated Li ions diffuse sluggishly and then are forced to deposit vertically and form an up‐and‐down surface. In the successive cycling, the Li dendrite is formed on the uneven surface along the protruding sites. On the contrary, the active Schottky defects in SDMECO@HINC modulation layer are capable of decreasing the desolvation energy barrier to generate bare lithium ions. Meanwhile, the reconstructed 4f‐center electrons by Schottky defects greatly facilitate a rapid Li transport for uniform Li nucleation and further lateral Li diffusion/plating to against the dendrite growth and volumetric expansion (Figure 5H), so that a very uniform surface with long lifespan is achieved.

### 2.5 Electrochemical Performances of Full Cells Based on SDMECO@HINC‐Li

**Figure 6:**
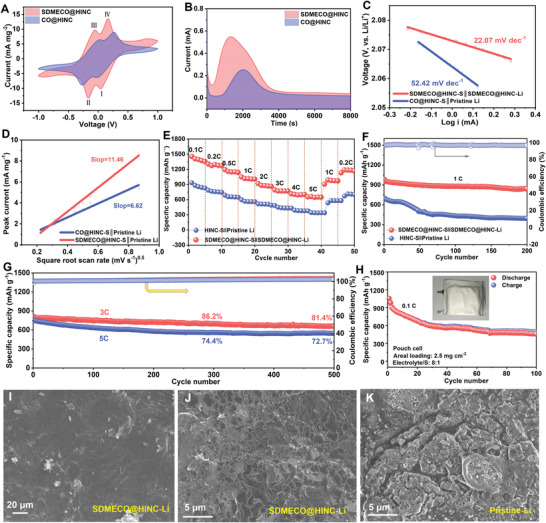
Electrochemical performances of full batteries based on SDMECO@HINC activator. A) Comparison of symmetric cells CV plots based on SDMECO@HINC and CO@HINC working electrode with a Li_2_S_6_ electrolyte at 10 mV s^−1^; B) the current response at the constant potential of 2.09 V in terms of a liquid/solid phase conversion threshold on different surface; C) Tafel plots derived from the second reduction peaks of the CV profiles; D) plot of the peak current recorded at the first anodic current peak (Li_2_S ↔ Li_2_S_4_) versus the square root of the scan rates; E) rate capability and F) cycling performances at current rate of 1 C for the two cells; G) long lifespan for 500 cycles measured at 3 and 5 C, respectively; H) cycle performance of SDMECO@HINC‐S║SDMECO@HINC‐Li pouch cell at 0.1 C; SEM images of the electrodes disassembled from the full battery at 1 C after 200 cycles: I,J) the exposed plated Li after peeling off the upper SDMECO@HINC layer in SDMECO@HINC‐S║SDMECO@HINC‐Li full battery under different magnification scales and K) the pristine Li in HINC‐S║pristine‐Li battery.

The catalytic functions of 4f‐electron modulated SDMECO@HINC is assessed in Li–S full battery. With the 4f‐center electronic interference introduced by Schottky defects, the interfacial electron structure is rebuilt to fertilize the adsorption centers and electrochemical catalytic efficiency toward sulfur species. Initially, the optical evolution images of SDMECO@HINC exhibits much stronger chemisorption activity to anchoring polysulfides (Figure S13, Supporting Information). As expected, the SDMECO@HINC catalyed electrode displays a much‐alleviated potential hysteresis (330 vs 538 mV) and higher conversion efficiency with lower charge transfer resistance (5 vs 11 Ω) (**Figure** **6**A; Figure S14, Supporting Information), indicating the rapid conversion of polysulfides. Meanwhile, a twice times higher peak current and earlier current response of Li_2_S precipitation on the SDMECO@HINC electrode is indicated in comparison to that of the controlled CO@HINC, implying the fast conversion and lower precipitation barrier of Li_2_S (Figure 6B). After sulfur incorporation (Figure S15, Supporting Information, XRD), the superiority of SDMECO@HINC in regulating the lithium kinetics is further demoed through assembling full battery. The SDMECO@HINC‐S║SDMECO@HINC‐Li full cell depicts lower Tafel slope (22.07 vs 52.42 mV dec^−1^), better peak reversibility, and lower charge transfer resistance (Figure 6C; Figures S16,S17, Supporting Information). Moreover, the Schottky defect‐rich full cell presents a higher lithium ion diffusion with the increased linear slope (11.46 vs 6.62), signifying better catalytic activity with catalytic SDMECO@HINC and lower conversion barriers to overcome (Figure 6D; Figure S18, Supporting Information).

Benefiting from the improved polysulfide conversion and Li kinetics, the SDMECO@HINC‐S║SDMECO@HINC‐Li full battery signifies the superior rate performance with a highly reversible capacity of 653 mA h g^−1^ and high CE close to 100% up to 5 C (Figure 6E; Figure S19, Supporting Information), much higher than the control one (355 mA h g^−1^). Two charge/discharge plateaus can still be observed with low overpotentials even at high rate of 5 C than the controlled HINC‐S║pristine Li cell (Figure S20, Supporting Information). Meanwhile, the full battery based on SDMECO@HINC in Figure 6F delivered a significantly strengthened capacity of 980 mA h g^−1^ at 1 C and higher reversible capacity after 200 cycles (840 vs 395 mA h g^−1^). As shown in Figure S21, Supporting Information, the cycling performance of the CO@HINC‐S║CO@HINC‐Li full cell is also exhibited. It is obviously observed that the CO@HINC modified full cell without sufficient 4f electron modulation presents much lower capacity and retention in comparison to the SDMECO@HINC regulated full cell, demonstrating that the improved performances are ascribed to the functions of tuning the 4f‐center electronic structures in the catalyst. It is well known that the lifespan at a high rate is quite critical for the future application attempt of batteries. As demonstrated in Figure 6G, the higher current rate at 3 and 5 C are further evaluated. In the long‐term cycles, both the cells based on SDMECO@HINC catalytic layer maintain high specific capacities and high capacity retentions as high as 81.4% and 72.7% at 3 and 5 C, respectively. After 500 cycles, the cell still preserves a low potential gap of ≈210 mV at 3 C and scarcely any voltage plateau degradation has been observed (Figure S22, Supporting Information). The pouch cell with areal sulfur loading of 2.5 mg cm^−2^ in electrolyte/sulfur ratio of 8:1 is attempted. As presented in Figure 6H, SDMECO@HINC‐S║SDMECO@HINC‐Li pouch cell achieves a high initial specific capacity of 1053 mA h g^−1^ at 0.1 C, contributing to the high energy density of 2264 W h kg^−1^ based on sulfur. After 100 cycles, it stabilizes the specific capacity at 1050 W h kg^−1^, suggesting the high reversibility. At last, the morphologies of both cycled SDMECO@HINC supported cathode and anode of the full battery are then observed, as exhibited in Figure 6I–K and Figure S23, Supporting Information. It is easy to figure out from Figure 6I,J that the as‐designed defect‐rich SDMECO@HINC layer is effective in kinetically guiding the Li atom deposition along the horizontal direction for build smooth and dendrite free interface at the anodic side to maintain the battery stability for long life‐span, in a sharp contrast to the ruptured and dendrite analogue of the pristine Li dissembled from the HINC‐S║pristine Li cell (Figure 6K). As a catalytic sulfur host, the defect‐rich SDMECO@HINC can beneficially catalyze the reaction kinetics to improve the high rate performance without cathodic structure crack (Figure 6E–G; Figure S23, Supporting Information).

## 3 Conclusions

In summary, a 4f‐center electron reconstruction strategy by forming Schottky defects is proposed to modulate lithium diffusion behaviors to suppress dendrites. As comprehensively confirmed, the modulated 4f‐center electronic density endows the catalyst with stronger capability in decreasing barriers of desolvation, nucleation, and diffusion for lateral and horizontal lithium deposition. Benefiting from electronic regulation, the SDMECO@HINC‐Li affords a long lifespan of 1200 h with lower overpotential of ≈13 mV and high Coulombic efficiency over 98%. The integrated Li–S full cells and pouch cell exhibit superior performance (653 mA h g^−1^ at 5 C) and excellent cycling stability at 3 C. This work provides a novel strategy to obtain long‐life lithium electrode by virtue of regulating electronic structures of activators.

## Conflict of Interest

The authors declare no conflict of interest.

## Supporting information

Supporting Information

## Data Availability

The data that support the findings of this study are available from the corresponding author upon reasonable request.
