# Supplementary material for: Tuning 4f‐Center Electron Structure by Schottky Defects for Catalyzing Li Diffusion to Achieve Long‐Term Dendrite‐Free Lithium Metal Battery
Source: Adv Sci (Weinh). 2022 Jun 8;9(23):2202244. doi: 10.1002/advs.202202244 (PMC9376855; doi:10.1002/advs.202202244)
Supplement: Supplementary file 1 — Supporting Information [file ADVS-9-2202244-s001.pdf]

Supporting information

Tuning 4f-center Electron Structure by Schottky Defects for Catalyzing Li Diffusion to Achieve Long-term Dendrite-free Lithium Metal Battery

Jing Zhang, Rong He, Quan Zhuang, Xinjun Ma, Caiyin You, Qianqian Hao, Linge Li, Shuang Cheng, Li Lei, Bo Deng, Xifei Li, Hongzhen Lin*, and Jian Wang**

Dr. J. Zhang, R. He, Prof. C. You, Q. Hao, Prof. L. Lei, Dr. B. Deng, Prof. X. Li
School of Materials Science and Engineering, Xi'an University of Technology, Xi'an 710048, China
E-mail: caiyinyou@xaut.edu.cn
L. Li, S. Cheng, Prof. H. Lin, Dr. J. Wang
i-Lab & CAS Key Laboratory of Nanophotonic Materials and Devices, Suzhou Institute of Nano-tech and Nano-bionics, Chinese Academy of Sciences, Suzhou, 215123, China
E-mail: hzlin2010@sinano.ac.cn; wangjian2014@sinano.ac.cn
Dr. J. Wang
Helmholtz Institute Ulm (HIU), Ulm D89081, Germany
E-mail: jian.wang@kit.edu
Dr. Q. Zhuang, Dr. X. Ma,
Inner Mongolia Key Laboratory of Carbon Nanomaterials, Nano Innovation Institute (NII), College of Chemistry and Materials Science, College of Mathematics and Physics, Inner Mongolia Minzu University, Tongliao 028000, China

Experimental Section

Synthesis of SDMECO@HINC nanocomposites: Carbon nanotubes (CNTs) powders (200 mg) and 1.1 mL polyethylene glycol were dispersed into 140 mL deionized water and then sonicated for 1 h to form homogeneous suspension. $Ce(NO)_3 \cdot 6H_2O$ crystalline powders of 40 mg were dissolved in the suspension with continuous stirring for 1 h. After that, about 3 mL ammonium hydroxide was dropwise added into the former mixture until the pH rising to eleven under continuous stirring for another 3 h. After sufficiently mixing, the above mixed dispersion was transferred into two 100 mL Teflon liner of stainless-steel autoclaves and placed in an oven at 120 ^{o}C keeping for 12 h. The resultant solid product was collected by filtration, washed by deionized water repeatedly, and later freezing dried for 24 h. Then, the Schottky defect-enriched SDMECO@HINC nanocomposite was finally obtained after annealing at 450 ^{o}C under Ar/H_2 (5%:95%, by volume) atmosphere for 2 h, with a heating rate of 5 ^{o}C min^{-1}. For comparison, the CeO_2 anchored highly-conductive and interconnected carbon nanotube networks (CO@HINC) were synthesized using the same method without the annealing step.

Preparation of sulfur cathodes nanocomposites: The sulfur cathodes nanocomposites were prepared through liquid loading method according to our previous works. [1,2] Typically, 200 mg of the above as-synthesized SDMECO@HINC was suspended in ultrapure water under sonication, then 21 mL Na_2Sx solution (1 mol L^{-1}) was added dropwise into the suspension under continuous stirring. Afterwards, 2 mol L^{-1} HCOOH solutions was added dropwise to the uniform blend for in-situ growth of sulfur on the SDMECO@HINC matrices under overnight continuous stirring. The resultant composites were collected, washed and freezing dried for 24 h, and finally sealed in a vessel full of argon gas. The vessel was heated at 155 °C for 12 h to efficiently infiltrate sulfur into the porous matrix to generate SDMECO@HINC-S composites. The CO@HINC-S composites were synthesized with the same method described above.

Catalysis related symmetric cell assembly: The electrochemical measurements were

conducted by assembling 2032 coin-type cells in an argon-filled glove box with pure Ar atmosphere. Firstly, 0.2 mol L^{-1} homogeneous Li_2S_6 solution is synthesized through dissolving Li_2S and sulfur in 1 mol L^{-1} lithium bis(fluorosulfonyl)imide (LiTFSI) in solvent of 1,2-dimethoxyethane (DME) and 1,3-dioxolane (DOL) in volume ratio of 1:1 via continuous stirring. Using SDMECO@HINC or CO@HINC electrodes as the cathode/anode separated by Celgard 2400 separator, the 2032-type symmetric cells were then assembled with addition of 40 μL of above polysulfide electrolyte in a glove box full of highly pure Ar gas. The Li_2S precipitation analysis was measured on the cells comprising of SDMECO@HINC or CO@HINC as cathode and commercial lithium metal as anode with 40 μL polysulfide electrolyte.

Li metal anode cell assembly: Before preparation of the modulation layer, 40 mg of SDMECO@HINC nanocomposite was dispersed into 80 mL ethanol under continuous sonication. The obtained dispersion was vacuum-filtered on the commercial separator with the areal loading of 0.18 mg cm^{-2}. Commercial Li foils were used without any pretreatment. In the Li-Li symmetric cell, the as-prepared SDMECO@HINC or the other thin layer was attached on the surface of commercial Li foils. In the Li-Cu asymmetric cell, the as-prepared SDMECO@HINC or the other thin film is covered on the surface of both the copper electrode and the coupled Li foil. 60 μL of the electrolyte (1 mol L^{-1} LiFSI dissolved in a mixed solvent of DME/DOL in volume ratio of 1:1) was added in each Li-Li and Li-Cu cell. For the tests of the Li-Li symmetric cells and Li-Cu asymmetric cells, the assembled batteries were precycled using a small current density for 10 times to stabilize the SEI formation and remove surface contaminations. In the stripping process, the cut-off voltage is set as 2.0 V versus Li/Li^+.

Li-S full cell assembly: To prepare sulfur cathodes, the obtained nanocomposites was fully mixed with carbon black and polyvinylidene fluoride (PVDF) binder (70:20:10 by weight) in an appropriate amount of N-methyl-2-pyrrolidone (NMP) solution via planetary mixer till forming uniform slurry. Then, the obtained slurry was pasted on aluminum foil using a doctor blade with a thickness of 150 μm (average sulfur loading of 1.5 mg cm^{-2}), which was followed by drying at 60 °C in a vacuum oven for

48 h. The working electrode was punched into discs of 11 mm in diameter for standby. The as-designed integrated Li-S full cells were assembled with SDMECO@HINC-S cathode, Celgard 2400 separator, and modified SDMECO @HINC-Li anode, using 1 M LiTFSI with 1 wt% $LiNO_3$ dissolved in mixed solvent of DME/DOL in volume ratio of 1:1 as the electrolyte (E/S of 15 μL mg^{-1}).

Materials and device characterization: The scanning electron microscopy (SEM) and transmission electron microscopy (TEM) of the samples were imaged using a Germany MERLIN compact scanning electron microscope (Zeiss Sigma HD) and a spherical aberration corrected scanning transmission electron microscope (JOEL NEO ARM200F) with an energy dispersive spectrometer, respectively. The X-ray diffraction (XRD) patterns were collected on an XRD-7000S X-ray diffractometer using Cu Kα radiation in a 2θ range from 10° to 80°. To determine the defects in the nanocomposites, the Raman spectra were recorded on a Horiba LabRAM ARAMIS spectrometer. The X-ray photoelectron spectra (XPS) were collected on an ESCALAB 250XI system to investigate the chemical surroundings of different elements in the nanocomposites. The sum frequency generation (SFG) test is performed on the commercial device. With the picosecond laser system, the visible light wavelength is fixed at 532 nm while the wavenumber of IR pulse is adjustable from 1000 to 4000 cm^{-1}. For the SFG measurements, the incident angle is 60° for the visible beam and 55° for the IR beam, thus the two lights directly shine on the electrode/solvent interface with/without SDMECO modulator. And then the sum frequency signal light is generated and reflected to the detector. The galvanostatic charge-discharge tests were conducted on a Neware Battery Testing System (BTS-5 V 20 mA) at various current rates. Electrochemical impedance spectroscopy (EIS) and cyclic voltammetry (CV) of coin cells were performed with a VMP-3 electrochemical working station.

Computational Details: The density functional theory (DFT) calculations were performed in the Vienna ab initio Simulation Package (VASP).[3] Interactions among the electrons and ions were simulated by the projector augmented-wave (PAW) method.[4] The Perdew-Burke-Ernzerhof (PBE) form of generalized gradient

approximation is adopted for the exchange-correlation energy functional,[5] and the cutoff energy was set to 400 eV. The Brillouin zones were sampled in a 3×3×1 Monkhorst-Pack k-mesh. Geometry optimization was repeated until the total energy change of each ionic step is less than 10^{-4} eV and the force on each atom is smaller than -0.03 eV/Å. The CeO_2 (111) slab was employed to simulate the interaction surface with Li atom. The modeled structures are large enough to avoid artificial interaction caused by periodicity. For all the spin-polarized periodic calculations, the Hubbard correction (DFT+U) is considered. [6] And a Hubbard U-like term was adopted to describe the onsite Coulombic interactions (Ueff = U-J). According to previous reports, the Ueff is 5.0 eV for Ce-4f electrons. We used the VASPKIT code for postprocessing of the VASP calculated data.[7]

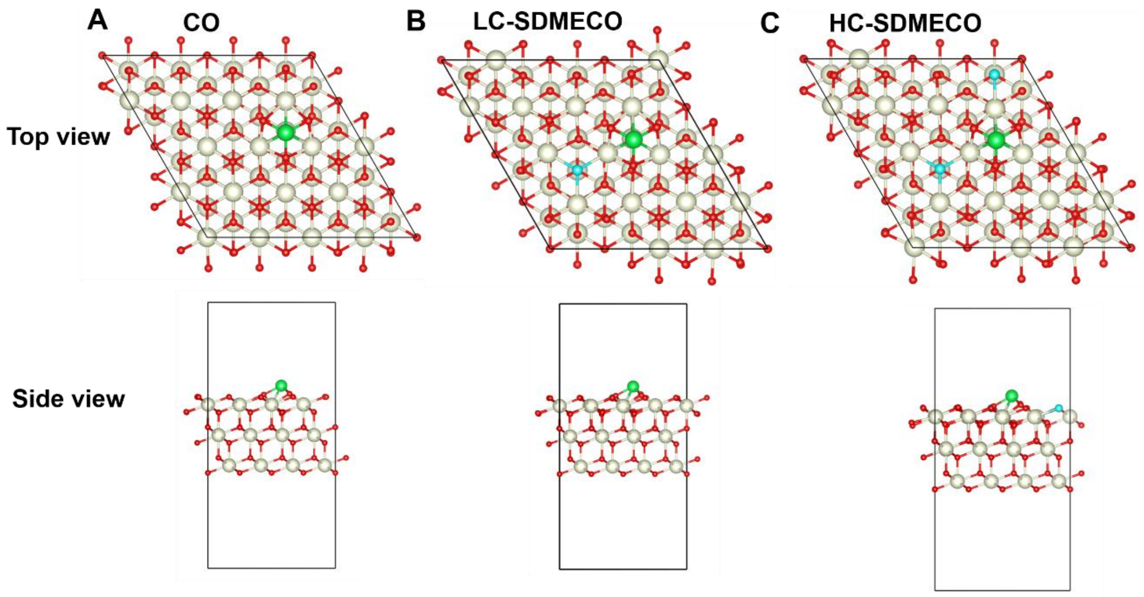

Figure S1 The top and side views of (A) CO-Li, (B) LC-SDMECO-Li and (C) HC-SDMECO-Li adsorption configurations (The white, green, and red balls represent the Ce, Li, and O atoms. And the deleted oxygen atoms for constructing the Schottky defects are depicted as cyan balls).

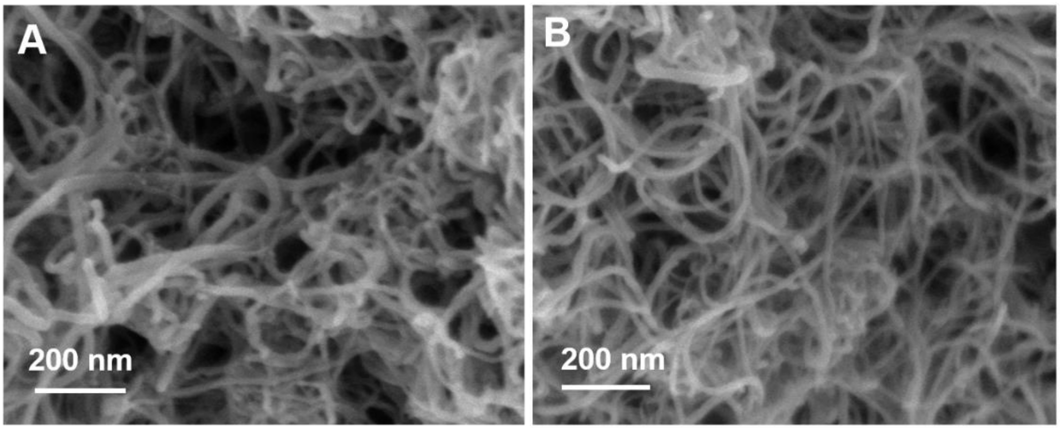

Figure S2 SEM images of (A) HINC and (B) CO@HINC.

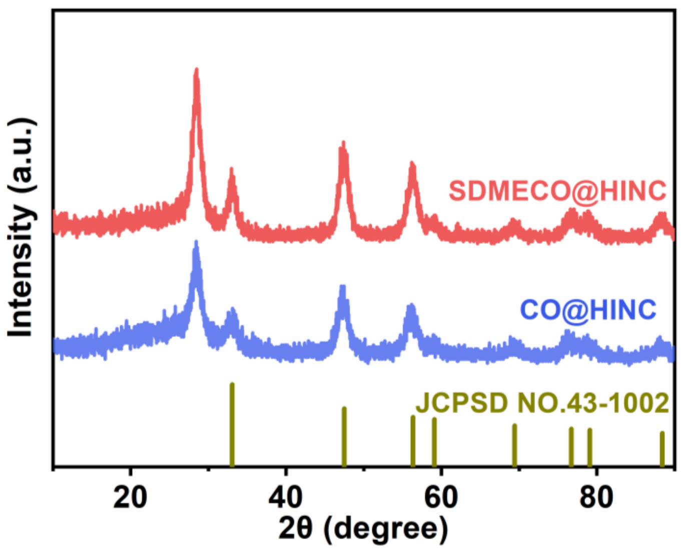

Figure S3 XRD spectra of the SDMECO@HINC and CO@HINC nanocomposites.

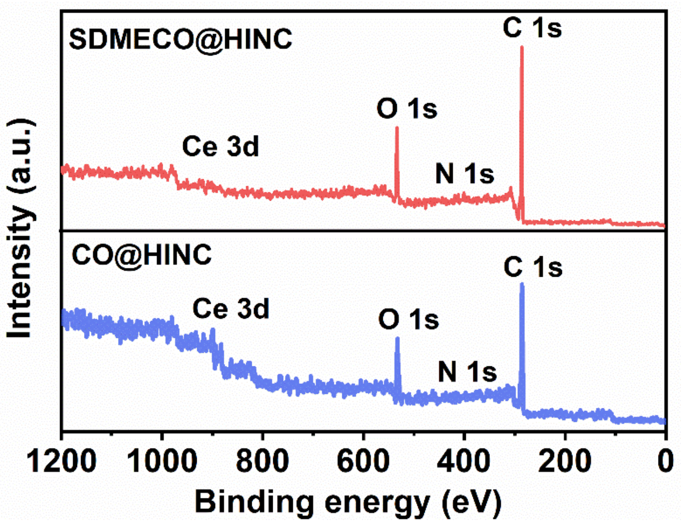

Figure S4 XPS full spectra of the SDMECO@HINC and CO@HINC nanocomposites.

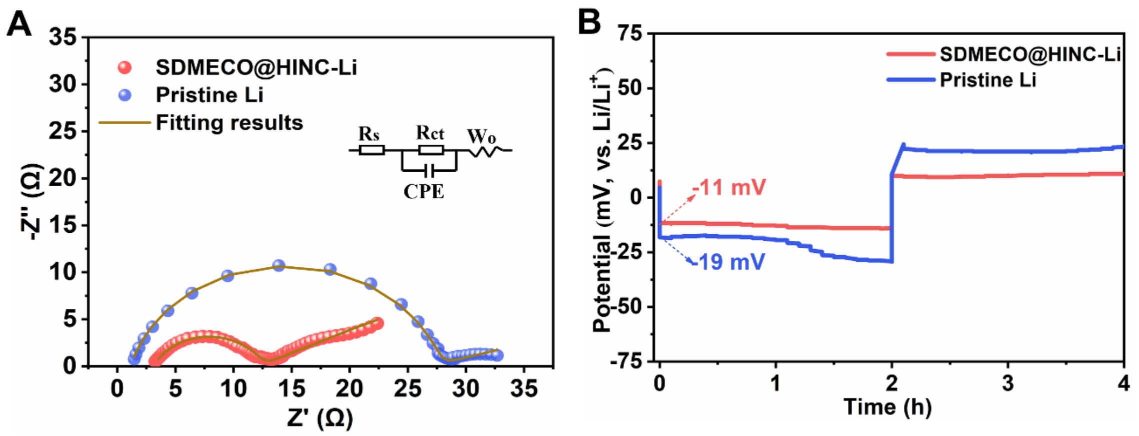

Figure S5 (A) EIS profiles on the defect induced SDMECO@HINC-Li and pristine Li symmetric cells; (B) Comparison of the Li nucleation barriers on different electrodes in symmetric cells at 0.5 mA cm^{-2} with capacity of 1 mA h cm^{-2}.

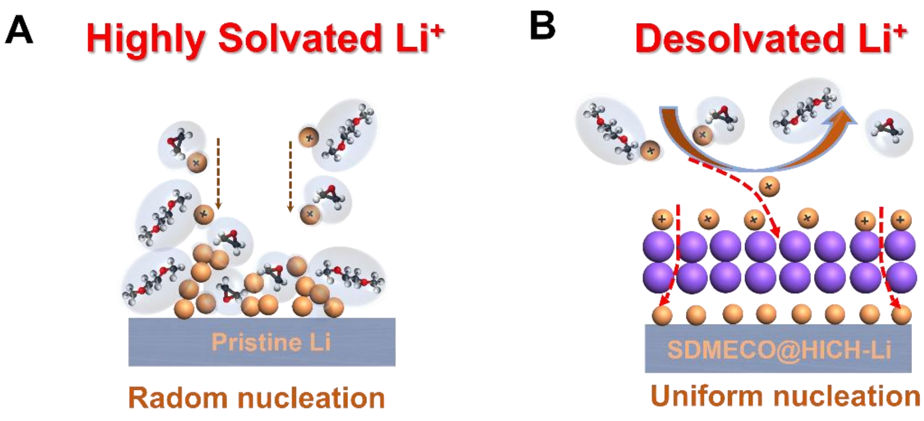

Figure S6 The schematic illustration of initial Li nucleation behaviors on (A) pristine Li and (B) SDMECO@HINC-Li in the early few cycles.

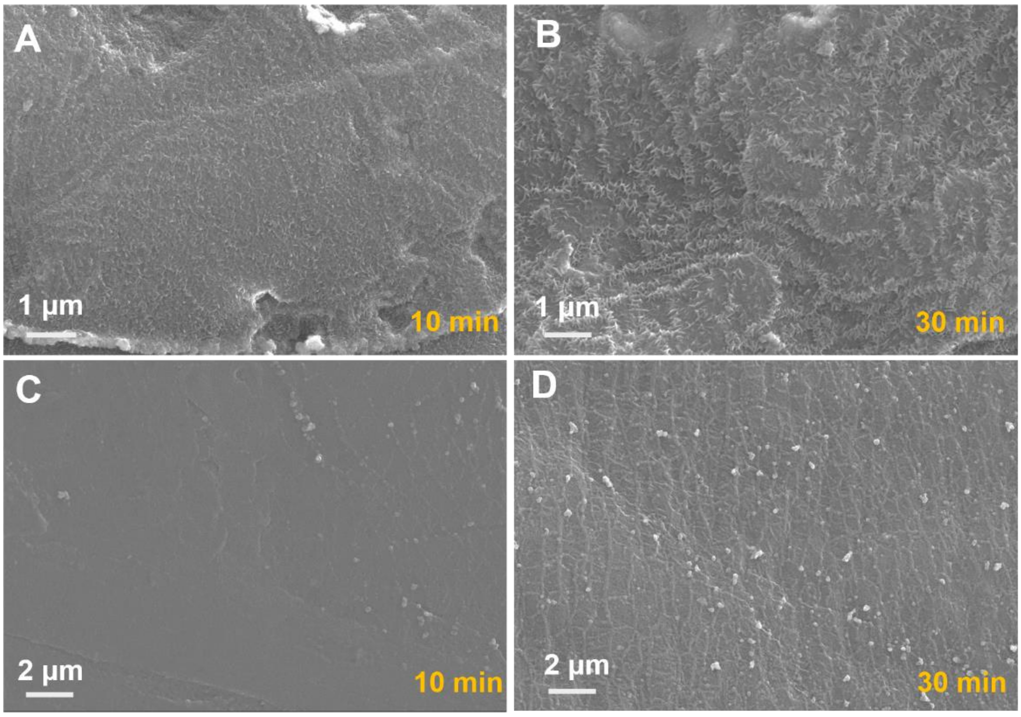

Figure S7 The top view SEM images of the nucleation process on (A), (B) bare Li surface and (C), (D) the exposed plated Li surface after uncovering the upper SDMECO@HINC layer for 10 min and 30 min, respectively.

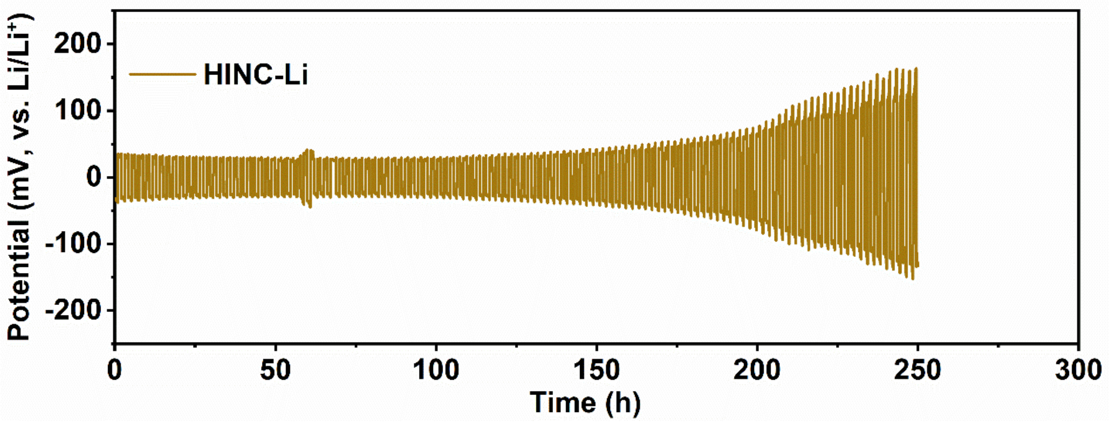

Figure S8 Voltage profiles of symmetric cells based on the HINC-Li electrodes under stripping/plating capacity of 1 mA h cm^{-2} at 1 mA cm^{-2}.

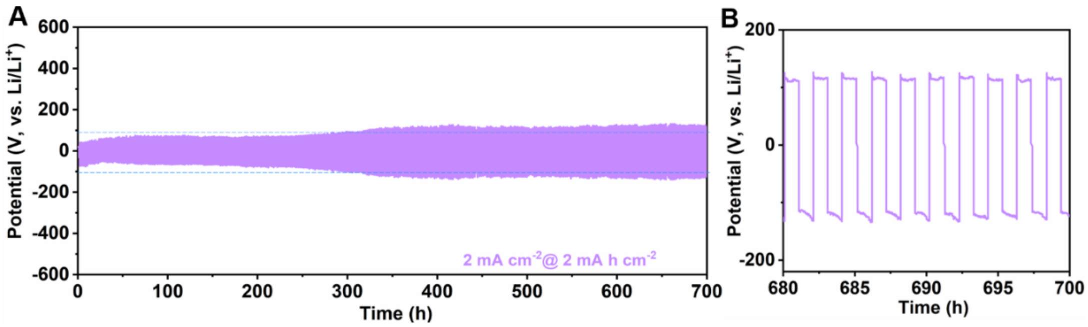

Figure S9 (A) and (B) Galvanostatic cycling of SDMECO@HINC-Li symmetric cells at 2 mA cm^{-2} under stripping/plating capacity of 2 mA h cm^{-2}.

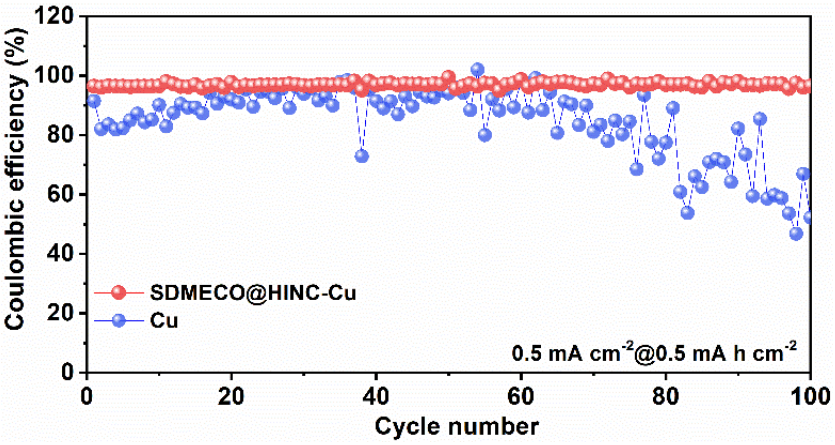

Figure S10 Coulombic efficiencies of Cu-Li cells based on the pristine Cu and SDMECO@HINC-Cu electrodes within 100 cycles under 0.5 mA cm^{-2}.

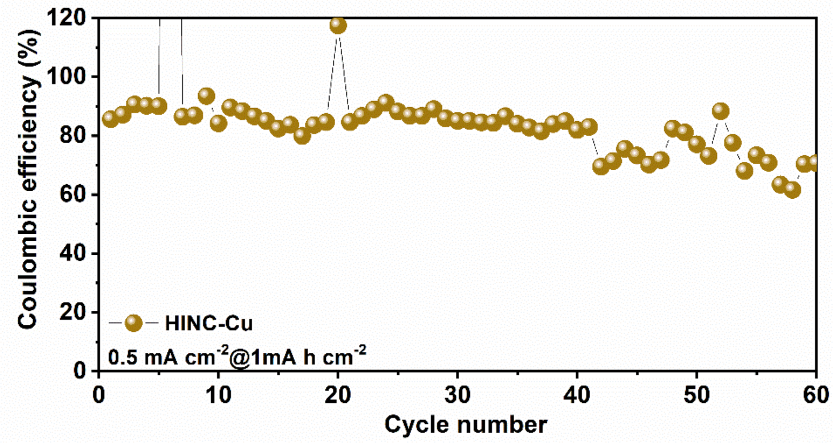

Figure S11 Coulombic efficiency of Cu-Li cells based on the HINC-Cu electrode within 60 cycles at 0.5 mA cm^{-2} under 1 mA h cm^{-2}.

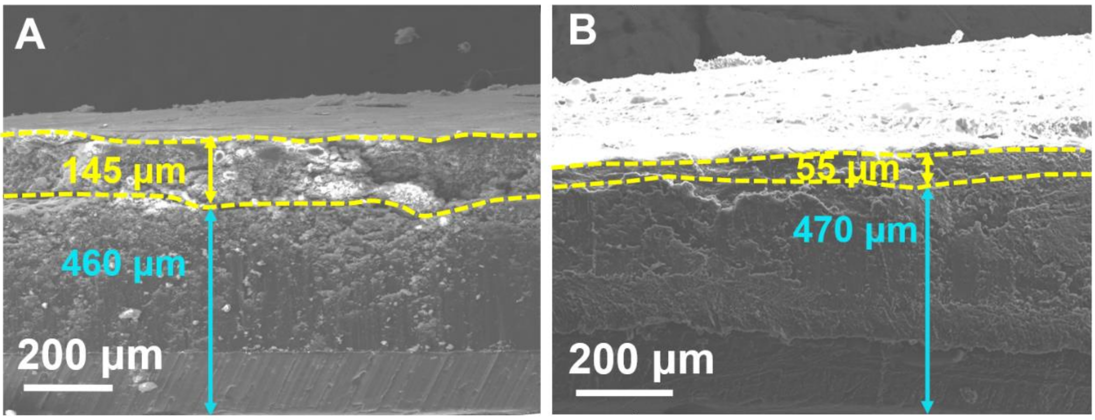

Figure S12 The cross-sectional SEM image of (A) the pristine Li and (B) the exposed plated Li surface after uncovering the upper SDMECO@HINC layer (Taking samples from the symmetric cells at the end of the cycling).

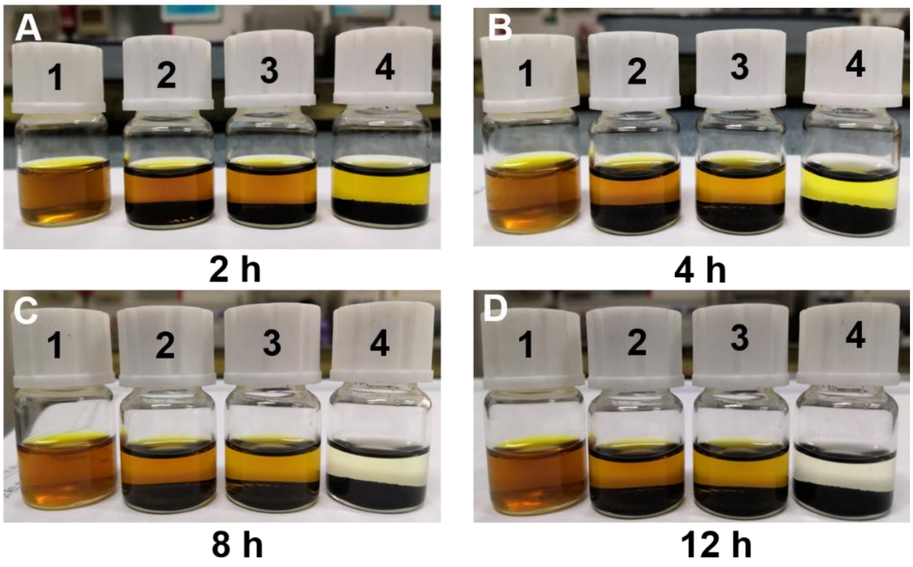

Figure S13 Digital photos of the SDMECO@HINC and the CO@HINC nanocomposites soaked in 5 mmol L^{-1} Li_2S_8 solution: after sufficient exposure to the adsorbents for (A) 2 h; (B) 4 h; (C) 8 h and (D) 12 h (Notably, No. 1, 2, 3, 4 represent the control, HINC, CO@HINC and SDMECO@HINC, respectively).

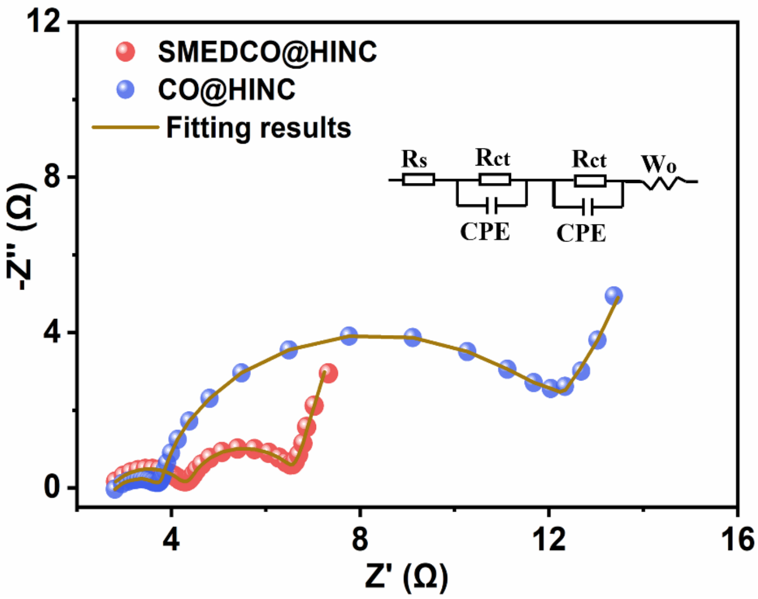

Figure S14 Comparison of EIS profiles carried out in polysulfide symmetric cell based pristine on SDMECO@HINC and CO@HINC electrodes.

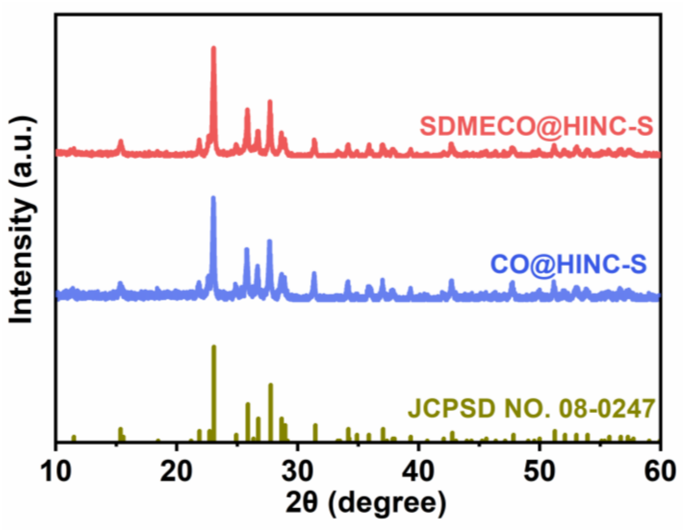

Figure S15 XRD spectra of the SDMECO@HINC-S and CO@HINC-S nanocomposites.

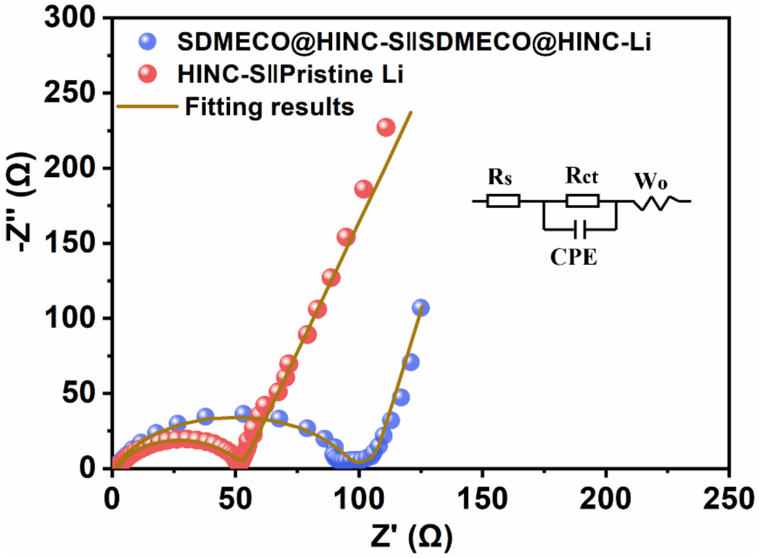

Figure S16 Comparison of EIS profiles carried out on SDMECO@HINC-S‖SDMECO@HINC-Li and CO@HINC-S‖Pristine Li full batteries.

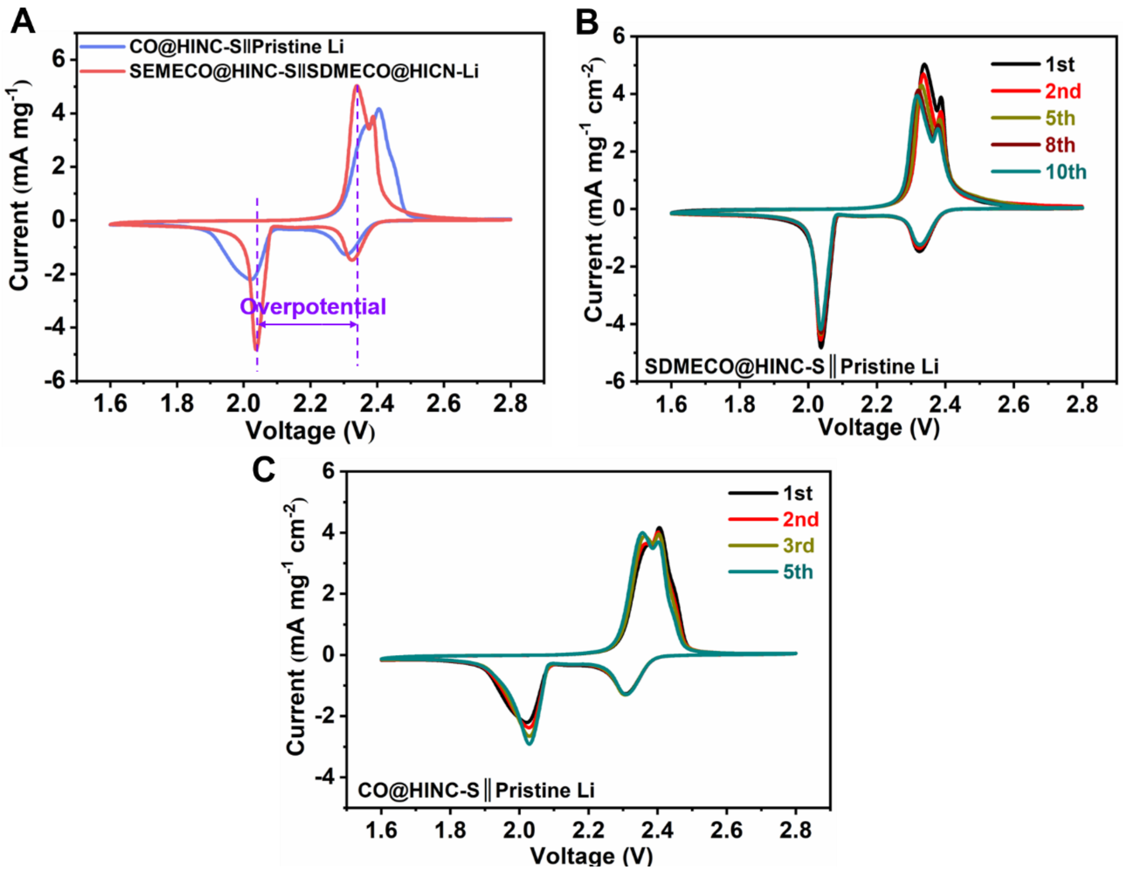

Figure S17 Cyclic voltammetry (CV) curves. Comparison of CV profiles at the first cycle at 0.1 mV s^{-1} within the scan range from 1.6 to 2.8 V (vs. Li/Li^{+}) in Li-S full batteries; The continuous scanning CV profiles of (B) SDMECO@HINC-S‖SDMECO@HINC-Li and (C) CO@HINC-S‖Pristine Li full batteries at the scan rate of 0.1 mV s^{-1}.

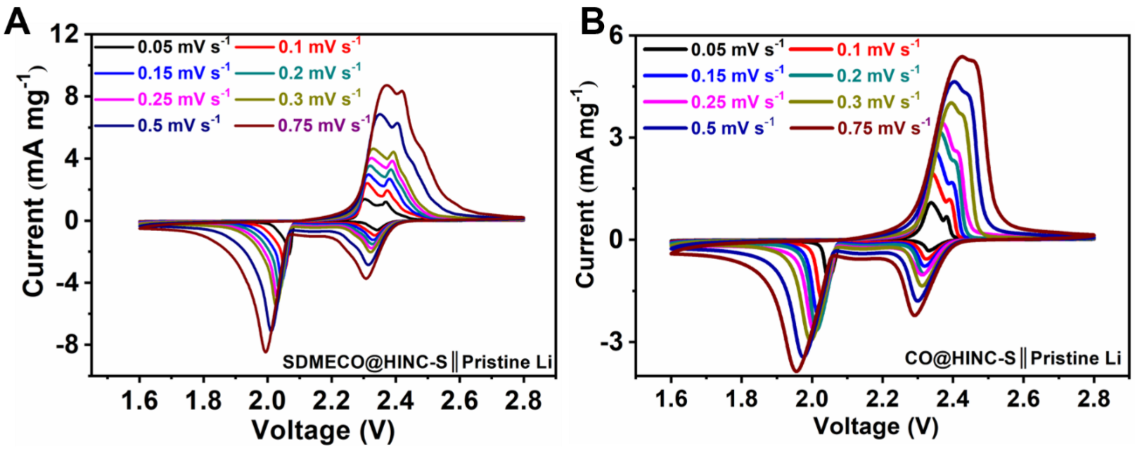

Figure S18 Scan rate-dependent CV profiles of (A) the SDMECO@HINC-S‖SDMECO@HINC-Li and (B) CO@HINC-S‖Pristine Li full batteries.

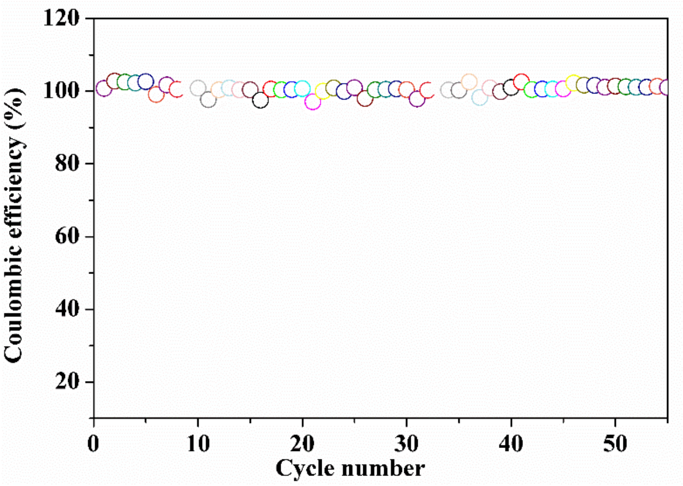

Figure S19 Coulombic efficiency of SDMECO@HINC-S‖SDMECO@HINC-Li full battery at incremental current rates.

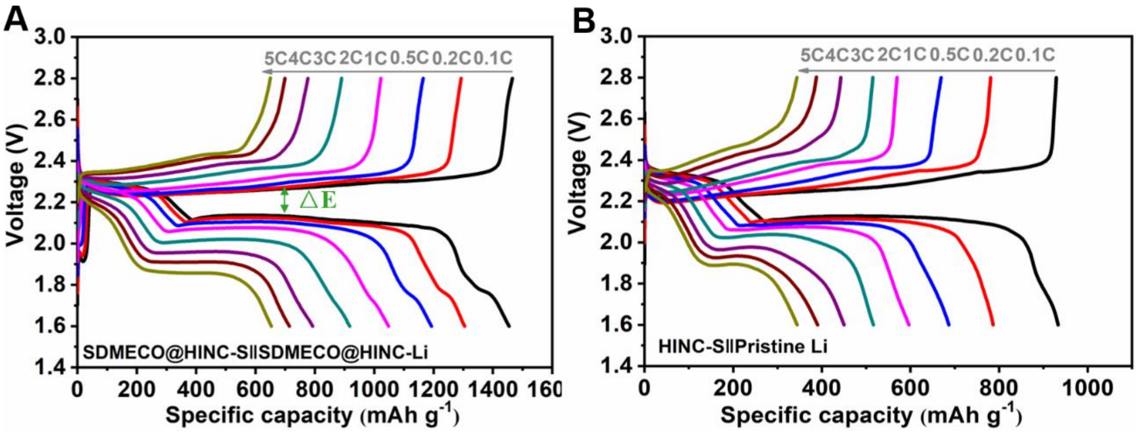

Figure S20 Galvanostatic charge and discharge curves for (A) SDMECO @HINC-S‖SDMECO@HINC-Li and (B) HINC-S‖Pristine Li cells at incremental current rates.

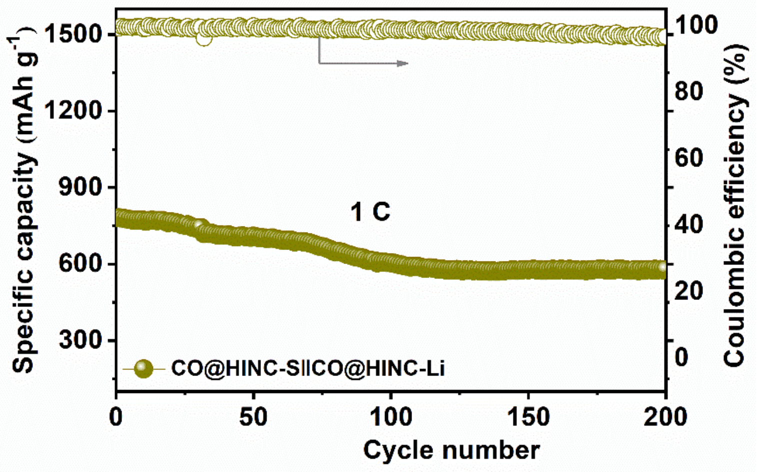

Figure S21 Cycling performance for the HINC-S‖HINC-Li full cell at 1 C.

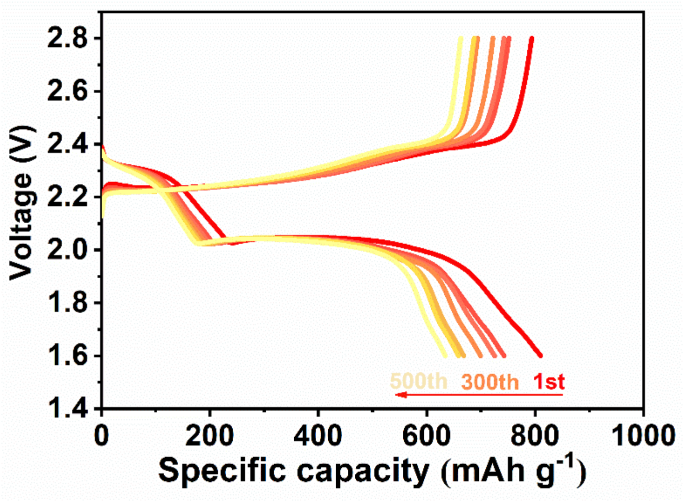

Figure S22 Galvanostatic charge and discharge curves obtained from the 1st to 500th cycles at 3 C for SDMECO@HINC-S‖SDMECO@HINC-Li full batteries.

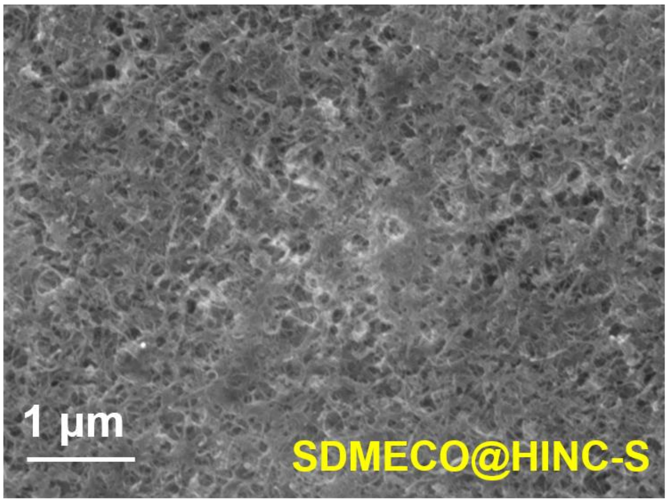

Figure S23 SEM images of the SDMECO@HINC-S cathode disassembled from the SDMECO @HINC-S‖SDMECO @HINC-Li full battery at 1 C for 200 cycles.

References

1. J. Zhang, S. Duan, C. You, J. Wang, H. Liu, S. Guo, W. Zhang, R. Yang, *J. Mater. Chem. A* **2020**, 8, 22240.
2. J. Zhang, C. You, J. Wang, H. Xu, C. Zhu, S. Guo, W. Zhang, R. Yang, Y. Xu, *Chem. Eng. J.* **2019**, 368, 340.

3 G. Kresse, J. Furthmüller, *Phys. Rev. B* **1996**, 54, 11169.

4 G. Kresse, D. Joubert, *Phys. Rev. B* **1999**, 59**,** 1758.

5 J. P. Perdew, K. Burke, M. Ernzerhof, *Phys. Rev. Lett.* **1996**, 77, 3865.

6 P. Mori-Sánchez, A. J. Cohen, W. Yang, *Phys. Rev. Lett.* **2008**, 100, 146401.

7 V. Wang, N. Xu, J.C. Liu, G. Tang, W.T. Geng, *Comput. Phys. Commun.* **2021**, 267, 108033.
